# Lithium Intoxication with Therapeutic Doses Following Laparoscopic Sleeve Gastrectomy: A Case Report and Review of the Literature

**DOI:** 10.1192/j.eurpsy.2024.762

**Published:** 2024-08-27

**Authors:** S. Kukurt, Z. Dönmez, O. Kilic, G. Dokuz, F. Coşkun, E. Yardımcı, I. Kırpınar

**Affiliations:** ^1^Bezmialem Vakif University, İstanbul, Türkiye

## Abstract

**Introduction:**

Lithium is a mood stabilizer often used as a first-line treatment for bipolar disorder. Its narrow therapeutic window and changes in the absorption, distribution, and elimination of the drug following bariatric surgery have important implications regarding patient safety.

**Objectives:**

We present a 51-year-old female patient with bipolar disorder and a medical history of morbid obesity, type 2 diabetes mellitus, hypothyroidism, hyperlipidemia, and essential hypertension. She was mentally stable on lithium 1200 mg/day, valproate 500 mg/day, and quetiapine 400 mg/day. She had undergone laparoscopic sleeve gastrectomy. After a month, she showed up to the emergency room (ER) with nausea, vomiting, diarrhea, and fatigue. Gastroenteritis was suspected until the patient started showing neurological symptoms such as delirium, dysarthria, ataxia, chorea, and athetosis.

**Methods:**

The patient was monitored and received aggressive intravenous hydration (3000 cc of 0.9% serum isotonic) in the intensive care unit (ICU). She was prescribed intramuscular biperiden injection of 5 mg/ml/day, pheniramine 45.5 mg/2 ml/day, and lorazepam 1 mg/day. Her lithium levels were checked every six hours. She was agitated and disoriented for the first five days despite lithium levels being in the therapeutic range. On day six, her blood lithium levels dropped to 0.399 mmol/L. Her psychiatric examination revealed that she resumed cooperation and orientation, her dysarthria subsided. However, her thought content and attitude were grandiose, and she had a labile affect. We prescribed 5 mg/day of olanzapine routinely and 1 mg/day of lorazepam on a needed basis. The next day, her labile affect became calmer, and her sleep improved so she was discharged from the ICU and admitted to general surgery inpatient service, and olanzapine was titrated to 10 mg per day since she had elevated mood symptoms.

**Results:**

After 7 days of intravenous hydration and supportive treatment, her neurological symptoms completely subsided except for a fine tremor, which lasted for another 3 days and then ceased. She was clinically stabilized without further need for intervention. Her lithium level was 0.206 mmol/L before her discharge.

**Conclusions:**

We believe it is of utmost importance to build a consensus in guidelines and inform physicians about lithium toxicity and its symptoms after bariatric surgeries. We recommend a careful follow-up of the patient pre-and postoperatively. Preoperative psychiatric intervention includes decreasing the lithium dose gradually and discontinuing it. After the operation, lithium can be started with a much lower dose and may be increased by checking lithium levels every week for at least 6 weeks after the operation until the patient can digest solid food again, and then every 2 weeks for 6 months, and thereafter every month for one year.

**Disclosure of Interest:**

None Declared

